# Treatment Outcome in Bilateral Cleft lip and Palate Patients Evaluated With the Huddart-Bodenham Scoring System and the Bilateral Cleft lip and Palate Yardstick: A Systematic Review

**DOI:** 10.1177/10556656211041883

**Published:** 2021-10-18

**Authors:** Wenying Kuang, Miranda Aarts, Anne Marie Kuijpers-Jagtman, Hong He, Edwin M. Ongkosuwito

**Affiliations:** 1 School & Hospital of Stomatology, 499766 Wuhan University, Wuhan, China; 2 Radboud University Medical Center, 6034 Radboud University, Nijmegen, the Netherlands; 3 University Medical Center Groningen, 10173 University of Groningen, Groningen, the Netherlands;; 4 School of Dental Medicine/Medical Faculty, 27210 University of Bern, Bern, Switzerland; Universitas Indonesia, Jakarta, Indonesia

**Keywords:** BCLP, dental arch relationship, Huddart-Bodenham scoring system, Bauru-BCLP yardstick, systematic review

## Abstract

**Objectives:**

To assess treatment outcome (transversal and sagittal dental arch relationships) and its determinants in complete bilateral cleft lip and palate (BCLP) evaluated with the modified Huddart-Bodenham scoring system and the BCLP Yardstick.

**Materials and methods:**

Multiple electronic databases were searched without time limitation. Randomized clinical trials, cohort and case control studies using BCLP Yardstick and/or modified Huddart-Bodenham system to judge treatment outcome of patients with BCLP were included. The Risk of Bias in Nonrandomized Studies of Interventions tool and Grading of Recommendations, Assessment, Development, and Evaluation was used.

**Results:**

Of the 528 studies identified by the electronic search, only eight retrospective studies met the inclusion criteria and were included. A total of 12 cleft centers were represented. All treatment protocols differed and background information was underreported. The results for the BCLP yardstick showed that all except the centers in New Zealand had a mean score lower than 3, indicating good treatment results. However, these studies had a moderate to high risk of bias. The modified Huddart-Bodenham scores were negative in all studies. No further meta-analysis was done due to heterogeneity and high risk of bias. The quality of evidence was graded as very low.

**Conclusion:**

Results for the dental arch relationship of studies in complete BCLP and possible determinants were not synthesized due to very low quality of evidence. Clinical research for patients with BCLP should focus on sound methodological designs to enable evidence-based decision making to improve treatment for patients with BCLP and thereby hopefully their quality of life.

## Introduction

Maxillofacial growth in patients with an orofacial cleft is influenced by intrinsic, functional, and extrinsic factors. The latter, in particular surgical interventions, may have a profound effect on maxillofacial growth. Different determinants may play a role like the surgeon, surgical technique, timing of the surgery and other possible factors (Shaw et al., 1992). To achieve an acceptable jaw relationship, good functional occlusion, and satisfactory dental and facial aesthetics, orthodontic and orthopedic treatments are indispensable (Kuijpers-Jagtman, 2012). Nowadays, there is still no agreement on the ideal treatment protocol leading to a wide diversity of treatment approaches (Shaw and Semb, 2017). Therefore, outcome studies are important to help clinicians to differentiate amongst protocols and select the optimal treatment for their patients.

Several tools have been developed to assess treatment outcomes in patients with orofacial clefts based on the assessment of the dental arch relationship which reflects the skeletal base relationship and may reflect the facial changes over time (Altalibi et al., 2013; Haque et al., 2015). One of the first tools developed to assess the dental arch relationship of patients with orofacial clefts was the Huddart-Bodenham system in 1972 (Huddart, 1972) that assesses arch form and occlusion. Several other tools were developed in the following decades, the most used are the Great Ormond Street London and Oslo (GOSLON) Yardstick (Mars et al., 1987) and the 5-year-old index (Atack et al., 1997) that assesses the anteroposterior dental arch relationship. However, all of these early tools were developed to judge treatment outcomes in patients with unilateral cleft lip and palate (UCLP) and not of patients with bilateral cleft lip and palate (BCLP), being the most severe phenotype in orofacial clefts (Tothill and Mossey, 2007). Its incidence is low compared to other phenotypes (Sivertsen et al., 2008) and it is for that reason that research in patients with BCLP is less common than in patients with UCLP (Ozawa et al., 2011). As a consequence, the first tool specially developed for patients with BCLP, the Bauru-Bilateral Cleft Lip and Palate Yardstick were only established in 2005 (Ozawa et al., 2005) later followed by the 6-, 9-, 12-Year-Olds’ BCLP yardsticks (Ozawa et al., 2011), while the modified Huddart-Bodenham system was firstly applied on patients with BCLP in 1997 (Heidbuchel and Kuijpers-Jagtman, 1997).

In contrast to UCLP (Nollet et al., 2005), no systematic review is available on treatment outcome for dental arch relationship for patients with BCLP. This systematic review was conducted to assess the transversal and sagittal dental arch relationship and its determinants in patients with complete bilateral cleft lip and palate evaluated with the modified Huddart-Bodenham scoring system and/or the BCLP Yardstick.

## Patients and Methods

### Protocol and Registration

The review protocol has been registered with the International Prospective Register of Systematic Reviews (PROSPERO) (registration number CRD42018108521). Data are reported according to the PRISMA guidelines (Preferred Reporting Items for Systematic Reviews and Meta-Analyses) (Liberati et al., 2009).

### Eligibility Criteria

Randomized clinical trials, cohort studies and case control studies using the BCLP Yardstick and/or the modified Huddart/Bodenham system (Huddart) to judge the treatment outcome of patients with BCLP were included in this review study. Type of participants were patients with nonsyndromic complete bilateral cleft lip and palate.

### Data Sources and Search Strategy

Studies were identified by searching electronic databases including Medline (Ovid), Embase (Ovid), CINAHL (EBSCO) and the Cochrane Library with no time limitation. Medical subject headings (MeSH) together with other related text words were combined to develop the search strategy (Table 1). No publication time was imposed. The last search was conducted on 7 July 2020. Additionally, expert-contact was employed as well as handsearching of references of included studies and conference abstracts.

**Table 1.** Multiple Database Search (Database of Medline, Embase, CINAHL, Cochrane Library) Until 8 Oct, 2018 (Updated on 7 July 2020).

Search Database(s)	Search No.	Search Criteria	No. Publications Found
Medline			
	1	Cleft Lip/ or Cleft Palate/ or cleft.ti,ab,kw. or cleft’.ti,ab,kw. or cleft's.ti,ab,kw. or clefted.ti,ab,kw. or cleftlip.ti,ab,kw. or cleftpalate.ti,ab,kw. or clefts.ti,ab,kw. or cheiloschi*.ti,ab,kw. or cheilognathoschi*.ti,ab,kw. or cheilognatoschi*.ti,ab,kw. or cheilognathopalatoschi*.ti,ab,kw. or cheilognatopalatoschi*.ti,ab,kw. or cheilopalatoschi*.ti,ab,kw. or palatoschi*.ti,ab,kw. or ([gnatho or palato] and [schisis or schizis]).ti,ab,kw. or BCLP.ti,ab,kw.	49 235
	2	(goslon or eurocran or bauru or yardstick or bodenham or huddart*).ti,ab,kw.	1039
	3	1 and 2	181
Embase			
	1	cleft lip palate/ or Cleft Lip/ or bilateral cleft lip/ or Cleft Palate/ or cleft.ti,ab,kw. or cleft’.ti,ab,kw. or cleft's.ti,ab,kw. or clefted.ti,ab,kw. or cleftlip.ti,ab,kw. or cleftpalate.ti,ab,kw. or clefts.ti,ab,kw. or cheiloschi*.ti,ab,kw. or cheilognathoschi*.ti,ab,kw. or cheilognatoschi*.ti,ab,kw. or cheilognathopalatoschi*.ti,ab,kw. or cheilognatopalatoschi*.ti,ab,kw. or cheilopalatoschi*.ti,ab,kw. or palatoschi*.ti,ab,kw. or ([gnatho or palato] and [schisis or schizis]).ti,ab,kw. or BCLP.ti,ab,kw.	55 395
	2	(goslon or eurocran or bauru or yardstick or bodenham or huddart*).ti,ab,kw.	1181
	3	1 and 2	209
CINAHL			
	1	(MH “Cleft Lip”# OR #MH “Cleft Palate”#	4659
	2	TI [cleft OR cleft’ OR cleft's OR clefted OR cleftlip OR cleftpalate OR clefts OR cheiloschi* OR cheilognathoschi* OR cheilognatoschi* OR cheilognathopalatoschi* OR cheilognatopalatoschi* OR cheilopalatoschi* OR palatoschi* OR [gnatho OR palato AND [schisis OR schizis]] OR BCLP] OR AB [cleft OR cleft’ OR cleft's OR clefted OR cleftlip OR cleftpalate OR clefts OR cheiloschi* OR cheilognathoschi* OR cheilognatoschi* OR cheilognathopalatoschi* OR cheilognatopalatoschi* OR cheilopalatoschi* OR palatoschi* OR [gnatho OR palato AND [schisis OR schizis]] OR BCLP]	6250

### Study Selection

All eligible articles were imported into the Covidence software (Covidence systematic review software, Veritas Health Innovation, Melbourne, Australia). Duplicate articles were removed by the system automatically. The inclusion criteria for eligible studies were the presence of one or more groups of patients with complete BCLP, using the BCLP Yardstick or the modified Huddart-Bodenham system (Huddart) to judge the dental arch relationship. The distribution of the scores should be present. Exclusion criteria were studies including only patients with UCLP, syndromic patients, narrative reviews and case reports, and no score distributions presented in the results. Titles, keywords and abstracts were screened by two examiners independently (WK, MA). In case of any disagreement between the examiners, disagreements were resolved by consensus after consultation of a third examiner (EO).

### Data Collection Process

The following information was extracted from each included study: first author, year of publication, country and city, number of patients, groups, and scoring system. As possible determinants for treatment outcome in BCLP the following data were extracted: gender, ethnic background, mean age of the patients at the time of dental arch relationship assessment, presence of Simonart's band or not, infant orthopedics, orthodontic treatment, treatment protocol, number of surgeons (Table 2).

**Table 2.** Background Information of the Eight Included Studies.

First author Year Country, city	Study group 1 *N*, sex, age (mean ± *SD*)	Study group 2 *N*, sex, age (mean ± *SD*)	Study group 3 *N*, sex, age (mean ± *SD*)	Study group 4 *N*, sex, age (mean ± *SD*)	Treatment period ethnicity	Syndrome Simonart's band	Outcome assessment	BCLP-Yardstick score	MHB score
Bartzela, 2010 *Sweden,* *Gothenburg* *Netherlands, Nijmegen* *Norway, Oslo*	*Gothenburg (G)*	*Nijmegen (N)*	*Oslo (O)*		Born before 1996 Caucasian	No associated congenital malformations, syndromes or mental retardations; Simonart's band included	BCLP-Yardstick		
	*N* = 56 F?, M? 6.82 ± 0.58 y	*N* = 37 F?, M? 6.02 ± 0.50	*N* = 107 F?, M? 6.02 ± 0.50					G: 2.42 ± 0.68 N: 2.20 ± 0.45 O: 2.26 ± 0.50	
	*N* = 50 F?, M? 9.68 ± 0.74 y	*N* = 42 F?, M? 8.95 ± 0.61	*N* = 112 F?, M? 6.02 ± 0.50					G: 2.27 ± 0.71 N: 2.26 ± 0.45 O: 2,43 ± 0.67	
	*N* = 40 F?, M? 12.92 ± 0.46 y	*N* = 40 F?, M? 12.26 ± 0.78	*N* = 101 F?, M? 6.02 ± 0.50					G: 2.49 ± 0.70 N: 2.72 ± 0.97 O: 2.41 ± 0.71	
Bartzela, 2011 *Netherlands, Nijmegen*	*N* = 37 F?, M? 6.09 ± 0.80 y				Born before 1996 Caucasian	No associated congenital malformations, syndromes, or mental retardations; Simonart's band included	MHB-scoring		−0.78 ± 0.48
	*N* = 41 F?, M? 8.95 ± 0.61 y								−0.74 ± 0.58
	*N* = 40 F?, M? 12.15 ± 0.89 y								−0.79 ± 0.57
Andlin Sobocki, 2012 *Sweden, Uppsala*	*Periosteoplasty*	*No-periosteoplasty*			pp: 1968–1977 no-pp: 1977–1985 Caucasian	No syndromes; Simonart's band included	MHB-scoring		pp −4.7 ± 7.2
	*N* = 15 pp F 6, M 9 16–19 y	*N* = 20 no-pp F 6, M 14 16–19 y							no-pp −1.7 ± 3.1
Dissaux, 2016 *France* *Strassbourg, Paris, Grenoble, Ecully (Lille)*	*Center A*	*Center B*	*Center C*	*Center D*	Period? Ethnicity?	No syndromes; Simonart's band included	BCLP Yardstick	Only percentages: Center A: 1 + 2, 0; 3, 50%; 4 + 5,50% Center B: 1 + 2, 12.5%; 3, 25%； 4 + 5， 62.5% Center C: 1 + 2, 20%; 3, 40%; 4 + 5,40% Center D: 1 + 2, 37.5%; 3, 25%; 4 + 5, 37.5%	
	*N* = 10 F?, M? 5.1 ± ? y	*N* = 10 F?, M? 4.9 ± ? y	*N* = 10 F?, M? 5.1 ± ? y	*N* = 10 F?, M? 5.8 ± ? y					
Cassi, 2017 *Italy, Parma*	*N* = 13 before ortho F 1, M 12 7.3 ± 3.7 y				Treated between 2004–2015	Syndrome? Simonart's band?	MHB-scoring		−10.7 ± 5.3
	*N* = 4 after ortho F 1, M 3 9.1 ± 1.9 y								Unknown
Batra, 2018 *India, Mount Abu, (Rajasthan)*	*N* = 50 F 22, M 28 12.52 ± 0.62 y				Born before 2005 Indian	No syndromes; Simonart's band?	BCLP-yardstick	2.34 ± 0.60	
Bittermann, 2018 *Netherlands, Utrecht*	*N* = 59, pre-ABG + premax ost F 22, M 37 10.34 ± 2.15 y				ABG + premax ost between 2004–2014 Ethnicity?	Syndrome? Simonart's band?	BCLP-Yardstick	2.31 ± 1.03	
	*N* = 59 post ortho F 22, M 37 14.33 ± 2.88 y							2.56 ± 1.33	
Fowler, 2019 New Zealand, *5 centers combined*	*N* = 32 F 13, M 19 9.9 ± 1.5 y				Born between 2000 and 2008 31% Maori 67% European	No syndromes; Simonart's band?	BCLP-Yardstick	3.38 ± 1.16	

### Risk of Bias of Individual Studies

The Risk Of Bias In Nonrandomized Studies of Interventions (ROBINS-I tool) (Sterne et al., 2016) was used as the assessment tool (Table 3). Included in the assessment were the following domains of bias: due to confounding, in the selection of participants into the study, in the classification of interventions, due to deviations from intended interventions, due to missing data, in the measurement of outcomes and in the selection of the reported result. Each study was judged by two examiners independently (WK, MA). Disagreements between the two examiners were resolved by consensus after consultation of a third examiner (EO).

**Table 3.** BCLP Treatment Protocols Based on Timing.

Timing	Center A (Gothenburg, Sweden)	Center B (Nijmegen, Netherlands)	Center C (Oslo, Norway)	Center D (Uppsala, Sweden)	Center E (Utrecht, Netherlands)	Center F (France)	Center G (France)	Center H (France)	Center I (France)	Center J (Parma, Italy)	Center K (Mount Abu, India)	Center L (New Zealand)
Birth	Infant orthopedics Duration 1.5 y Nose plugs Duration 2.5 y	Infant orthopedics (plate + extra-oral strapping Mean duration 9.2 mo			Infant orthopedics [plate] on indication Age few wks		Dental arch relationship outcomes for children with complete unilateral and complete bilateral cleft lip and palate in new zealand.					
2 months							Infant orthopedics [active plate]					
3 months	Bilateral lip adhesion Mean age 3.3 mo		Straight-line lip repair + hard palate repair on one side Mean age 3.4 mo	1st side lip repair [Skoog's technique] Mean age 3 months + infant periosteoplasty [in BCLP-pp group]		Lip repair [Millard]	Lip adhesion + straight veloplasty	Intravelar veloplasty				Lip repair at 3–6 mo of age
5 months			Straight-line lip repair + hard palate repair on the other side Mean age 4.9 mo					1st side lip repair + homolateral hard palate repair [Malek technique]				
6 months		One-stage lip repair [modified Manchester] Mean age 7.2 mo		2nd side lip repair [Skoog's technique] Mean age 6 mo		Palate repair [Veau-Wardill flaps] + straight veloplasty Age 6–8 mo	Lip repair [Millard] + hard palate repair using free tibial periosteum graft [Stricker] Age 6–8 mo	2nd side lip repair + hard palate repair [Malek technique] Age 6–8 mo	Millard Lip repair + primary septorhinoplasty [Millard modified Talmant technique] + intravelar veloplasty [Sommerlad] Age 6–8 mo		Lip repair [Millard] Mean age 6.8 mo	
9 months	Soft palate repair [center's own technique] Mean age 8.5 mo			Soft palate repair [BCLP-np group] Mean age 9 mo								Palate repair at 9–12 mo
12 months		Soft palate repair [Modified Von Langenbeck] Mean age 13.8 mo			Lip repair first year of life [modified Millard or Tennison technique], Lip adhesion for wide clefts, before primary lip repair Soft palate repair [opposing Z-plasty + union of the M. levator veli palatini] Mean age 1 y				Hard palate repair without raising flaps [Talmant] Age 12–14 mo			
18 months	Definitive bilateral lip and nose repair [center's own technique] Mean age 18 mo		Soft palate repair [Von Langenbeck] Mean age 19 mo	One-stage palate repair for BCLP-pp group [modified Veau-Wardill method] Age 20–40 mo Hard palate repair for BCLP-np group Age 20–48 mo							Palate repair [pushback technique] Mean age 18 mo	
4–6 years		Hard palate repair [Von Langenbeck] [before 1975] Mean age 3.8 y			Hard palate repair [von Langenbeck]Mean age 6 y Pharyngoplasty at young age if early speech development was inadequate							
9 years	One-side alveolar bone grafting [tibia] Mean age 8.0 y Hard palate closure with alveolar bone grafting of second side Mean age 8.5 y	Hard palate repair and bilateral alveolar bone grafting [chin; after 1975] + osteotomy of premaxilla Mean age 9.9 y	Bilateral alveolar bone grafting [iliac crest] Mean age 9.9 y	Transverse and/or sagittal expansion before bone grafting Bone grafting in BCLP-np group [iliac crest] with one alveolar cleft at a time and with 6 mo apart) Mean age 10.5 y	Preoperative orthodontic alignment (over 90% patients) Closure of the alveolar process (premaxilla osteotomy) Age 8–12 y					Maxillary expansion/ Maxillary protraction/ Incisor alignment and proclination Mean age 7.3 ± 3.7 y		
12 years				Transverse and/or sagittal expansion before bone grafting Bone grafting in BCLP-pp group (iliac crest) with one alveolar cleft at a time and with 6 mo apart) Mean age 11.5 y								
Notes	No information on orthodontic treatment	No information on orthodontic treatment	No information on orthodontic treatment	Both groups received orthodontic treatment with transverse and/or sagittal expansion, mainly with removable appliances in the mixed dentition before bone grafting and in the permanent dentition with fixed appliances	Postoperative fixed appliance for aligning the permanent dentition and moving the canine or lateral incisor into the bone grafts	No orthodontic treatment before evaluation	No orthodontic treatment before evaluation	No orthodontic treatment before evaluation	No orthodontic treatment before evaluation	No information on surgical protocols	No orthodontic treatment before evaluation	No orthodontic treatment was done before evaluation

### Certainty of the Evidence

The GRADE tool (Grading of Recommendations, Assessment, Development, and Evaluation) was used to assess the quality of a body of evidence (GRADE working group). The quality of evidence was rated per outcome into one of four categories (high, moderate, low, very low).

### Summary Measures and Synthesis of Results

All outcome data are reported as differences of means (mean and standard deviations). Depending on the homogeneity of the included studies, a quantitative analysis will be carried out, otherwise, a narrative synthesis will be given.

## Results

### Study Selection

**Figure 1.**
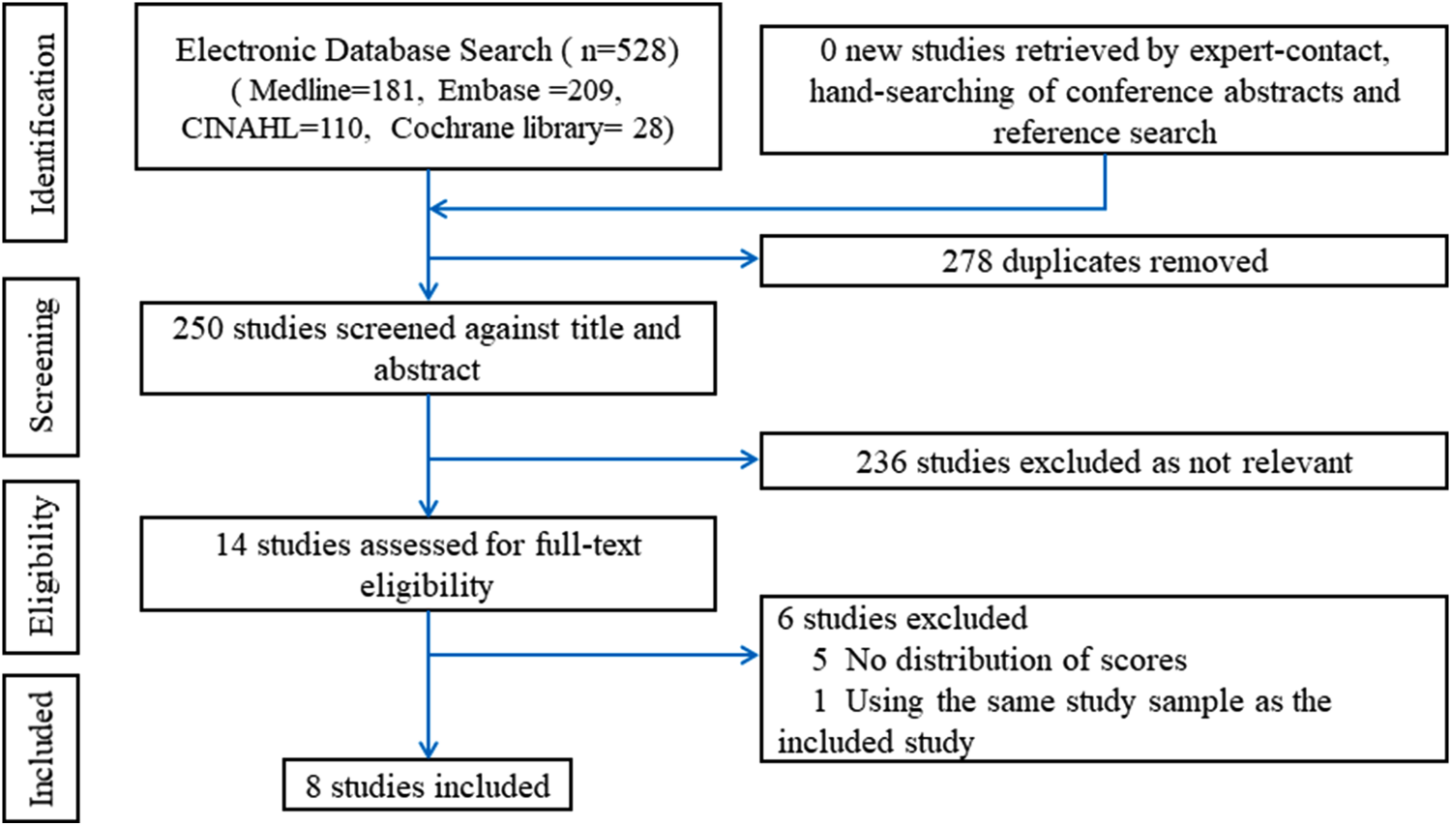
PRISMA flow diagram.

Abbreviation: PRISMA, Preferred Reporting Items for Systematic Reviews and Meta-Analyses.

In total, 528 studies were identified by the electronic search: 181 from Medline, 209 from Embase, 110 from CINAHL, and 28 from the Cochrane library. No other studies were retrieved by expert contact or hand-searching. 278 studies were excluded due to duplication, 236 studies were discarded as not relevant after the title and abstract screening. The remaining 14 studies were assessed for full-text eligibility, and five of them were excluded due to the lack of distributions of the scores (Tothill and Mossey, 2007; Ozawa et al., 2011; Leenarts et al., 2012; Dobbyn et al., 2015; Ma et al., 2017) and one (Thompson et al., 2019) for using the same study sample as another included study. The remaining eight studies were included in the systematic review (Bartzela et al., 2010, 2011; Andlin Sobocki et al., 2012; Dissaux et al., 2016; Cassi et al., 2017; Batra et al., 2018; Bittermann et al., 2018; Fowler et al., 2019). The PRISMA flow diagram is presented in Figure 1.

### Study Characteristics

The included studies and their characteristics are presented in Table 2. All eight included studies were retrospective studies (Bartzela et al., 2010, 2011; Andlin Sobocki et al., 2012; Dissaux et al., 2016; Cassi et al., 2017; Batra et al., 2018; Bittermann et al., 2018; Fowler et al., 2019), three of them were intercenter outcome studies (Bartzela et al., 2010; Dissaux et al., 2016; Fowler et al., 2019), the other five were intracenter studies (Bartzela et al., 2011; Andlin Sobocki et al., 2012; Cassi et al., 2017; Batra et al., 2018; Bittermann et al., 2018).

In total, 16 centers were involved, but Fowler et al. (2019) presented only combined data of the five centers in New Zealand as similar surgical protocols were used, and therefore we considered these 5 centers as one center. So we ended up with 12 centers. We re-named the 12 centers: Center A, Gothenburg, Sweden; Center B, Nijmegen, the Netherlands; Center C, Oslo, Norway; Center D, Uppsala, Sweden; Center E, Utrecht, the Netherlands; Center F, France; Center G, France; Center H, France; Center I, France; Center J, Parma, Italy; Center K, Mount Abu, India; Center L, New Zealand. The treatment protocols of these centers are summarized in Table 4 based on the timing of the procedures. One study [Bartzela et al., 2011] presented the BCLP yardstick scores at 9 years of age of 42 patients with BCLP from Nijmegen, the Netherlands, which were also presented in another study [Bartzela et al., 2010]. We took the MHB-scores from [Bartzela et al., 2011] and used the BCLP yardstick scores from [Bartzela et al., 2010], as the latter study compared the BCLP yardstick scores of three different centers.

**Table 4.** Results of the Risk of the Bias Assessment.

Domain	Bartzela, 2010	Bartzela, 2011	Andlin Sobocki, 2012	Dissaux, 2016	Cassi, 2017	Batra, 2018	Bittermann, 2018	Fowler, 2019
Bias due to confounding	Moderate	Moderate	Moderate	Moderate	Moderate	Moderate	Moderate	Moderate
Bias in selection of participants into the study	Low	Low	Moderate	Low	Serious	Low	Moderate	Low
Bias in classification of interventions	Low	Low	Low	Low	Low	Low	Low	Low
Bias due to deviations from intended interventions	Low	Low	Low	Low	Low	Low	Low	Low
Bias due to missing data	Low	Low	Low	Low	Critical	Low	Moderate	Moderate
Bias in measurement of outcomes	Low	Low	Serious	Low	Moderate	Low	Low	Low
Bias in selection of the reported result	Low	Low	Low	Moderate	Serious	Low	Low	Low
Overall*	Moderate	Moderate	Serious	Moderate	Critical	Moderate	Moderate	Moderate

Overall Bias ( instead of just "overall")

The gender distribution was only mentioned in five studies (Andlin Sobocki et al., 2012; Cassi et al., 2017; Batra et al., 2018; Bittermann et al., 2018; Fowler et al., 2019), but in all studies males and females were analyzed as one group. The ethnical background was reported in five studies with three being Caucasian (Bartzela et al., 2010, 2011; Andlin Sobocki et al., 2012), one Indian (Batra et al., 2018), and one with Maori and European ethnic background (Fowler et al., 2019); Two studies did not mention ethnicity (Cassi et al., 2017; Bittermann et al., 2018) and one study just stated that the included patients were homogeneous (Dissaux et al., 2016).

The included patients of six studies were all nonsyndromic (Bartzela et al., 2010, 2011; Andlin Sobocki et al., 2012; Dissaux et al., 2016; Batra et al., 2018; Fowler et al., 2019). However, two studies did not mention whether the included patients were syndromic or not (Cassi et al., 2017; Bittermann et al., 2018). None of the treatment protocols of the included 12 centers were similar (Table 4).

To judge the dental arch relationship, two studies used the Huddart scoring system (Andlin Sobocki et al., 2012; Cassi et al., 2017), five studies used the BCLP Yardstick (Bartzela et al., 2010; Dissaux et al., 2016; Batra et al., 2018; Bittermann et al., 2018; Fowler et al., 2019), and one study used both (Bartzela et al., 2011).

### Risk of Bias Within Studies

The results of the risk of bias assessment are presented in Table 5. As stated in the detailed guidance for the ROBINS-I tool (Sterne et al., 2016), when “confounding is expected, and all known important confounding domains are appropriately measured and controlled for”, the study can be seen as having a moderate bias in the domain of bias due to confounding. All the included studies were judged as having a moderate risk of bias due to confounding. The age of patients at the time of dental arch relationship judgement was considered as the most important confounding domain, and it was appropriately controlled for in each study, however, these studies are not comparable to a well-performed randomized trial regarding confounding. Finally, the overall risk of bias was for six of the eight included studies considered as having a moderate risk of bias (Bartzela et al., 2010, 2011; Dissaux et al., 2016; Batra et al., 2018; Bittermann et al., 2018; Fowler et al., 2019). Two studies were considered as having serious (Andlin Sobocki et al., 2012) or critical (Cassi et al., 2017) risk of bias.

**Table 5.** Results of Certainty of the Evidence (GRADE Assessment).

	Quality assessment						
No. of studies	Design	Limitations	Inconsistency	Indirectness	Imprecision	Other considerations	Overall score
Dental arch relationship assessed by MHB							
3	Observational studies	Very serious	Serious	No serious indirectness	Very serious	None	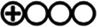
Dental arch relationship measured with BCLP yardstick							
5	Observational studies	Serious	Serious	No serious indirectness	Very serious	None	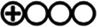

4 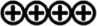 **High **=  This research provides a very good indication of the likely effect. The likelihood that the effect will be substantially different** is low.

3 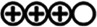 **Moderate** =  This research provides a good indication of the likely effect. The likelihood that the effect will be substantially different** is moderate.

2 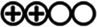 **Low** =  This research provides some indication of the likely effect. However, the likelihood that it will be substantially different** is high.

1 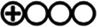 **Very low** =  This research does not provide a reliable indication of the likely effect. The likelihood that the effect will be substantially different** is very high.

### Results of Individual Studies

The dental arch relationship judgment results of individual studies are listed in Table 2. In the studies using the modified Huddart-Bodenham scoring system to judge the treatment outcome, Andlin Sobocki et al. (2012) found that in the BCLP-pp group [patients treated with periosteoplasty], the anterior, buccal right side, buccal left side and the total score all had a more negative crossbite score than the corresponding segment in the BCLP-np group [patients that had no periosteoplasty but secondary bone grafting] at 16–19 years of age. In the study of Cassi et al. (2017), only the preorthodontic treatment [T0] HB total score in patients with BCLP was presented [−10.7 ± 5.3]. Amongst the studies using the BCLP Yardstick to judge the treatment outcome, Bartzela et al. (2010) compared the dental arch relationship in patients with BCLP treated by three different centers [Center A, Gothenburg, Sweden; Center B, Nijmegen, Netherlands; Center C, Oslo, Norway], the mean score for the 6-year group was significantly lower [more favorable] in center B than in center A. Among the 9 and 12-year groups, there were no significant differences in the mean scores between the three centers. Batra et al. (2018) compared the BCLP yardstick scores of the 12-year group of their cleft center with the three centers mentioned in the research of Bartzela et al., the mean BCLP yardstick score for their center was 2.34 ± 0.60. Bittermann et al. (2018) also compared their treatment outcome with the three centers mentioned above, and the pre-SABG [secondary alveolar bone grafting] [9-year group] mean BCLP yardstick score was 2.31 ± 1.03, the end-point [12-year group] mean BCLP yardstick score was 2.56 ± 1.33, while no statistical difference was found in the mean BCLP-score among the four centers. Dissaux et al. (2016) compared the treatment outcome of 5-year-old children with BCLP among 4 centers in France, but only the percentage of the patients with BCLP-yardstick scores 1 or 2, 3, 4 or 5 were presented. Of the four centers, Center D had the highest percentage of better treatment outcomes [1 + 2], with a rate of 37.5%. Bartzela et al. (2011) compared the MHB-scoring system and the BCLP Yardstick scoring for the evaluation of treatment outcome in patients with BCLP, and the mean MHB-score was negative for all dental arch segments. The values of the two scoring systems showed a highly significant negative correlation for all teeth and all ages. Fowler et al. (2019) reported an inferior dental arch result in 9-year-old children compared to other studies: in the included 32 CBCLP patients, 7 [21.9%] were “very good/good” [Grade 1 *N* = 1; Grade 2 *N* = 6], *N* = 13 [40.6%] were “fair” [Grade 3] and *N* = 12 [37.5%] were “poor/very poor” [Grade 4 *N* = 4; Grade 5 *N* = 8].

### Synthesis of Results

In the included studies, 12 centers from seven countries were represented and all treatment protocols were different (Table 4). Gender distribution, ethnical background, and presence/ absence of a syndrome were underreported. Furthermore, the risk of bias analysis showed that six of the eight included studies were considered as having a moderate risk of bias and the other two studies were considered as having a serious/critical risk of bias. Therefore, due to high heterogeneity and risk of bias the data cannot be used for a further meta-analysis.

### Certainty of the Evidence

The certainty of the evidence was assessed using the GRADE tool. Observational studies start as low quality of evidence. Due to limitations in the studies the quality of evidence was assessed as very low (Table 5). This means that we have very little confidence in the effect estimate: the true effect is likely to be substantially different from the estimate of effect.

## Discussion

This systematic review investigated the dental arch relationship in patients with a complete BCLP. Unfortunately, we were not able to synthesize the results of the included studies into a meta-analysis because of the heterogeneity of the included studies. A total outcome score for dental arch relationship and the effect of different background variables could therefore not be determined. The certainty of the evidence of the two outcomes that were measured is very low.

To compare treatment outcomes in patients with oral cleft, the high variability in background variables should be overcome or dealt with in a methodological sound way. Each included study reported on a different combination of background variables (Bartzela et al., 2010, 2011; Andlin Sobocki et al., 2012; Dissaux et al., 2016; Cassi et al., 2017; Batra et al., 2018; Bittermann et al., 2018; Fowler et al., 2019)*.* Ethnicity, gender, the same and correct phenotype, year of birth, age at outcome and consecutiveness of cases should be reported on and methodologically taken into account and in a study using a dental related classification the dental developmental stage should also be included. Other influencing factors such as number of surgeons, surgical technique, number, timing, revisions and complications of surgeries, number of orthodontists, presurgical orthopedics, orthodontic expansion, or other orth treatment and whether a standardized protocol was used should be included in the reporting. According to the protocol used by a center even other influencing factors should be taken into account differing amongst protocols and this makes treatment outcome comparison amongst centers a hardship. The existence of many different treatment protocols was already reported in 2000 (Shaw et al., 2001). Still many different treatment protocols are used nowadays and in the 12 included centers in this study, none of the 12 centers used the same protocol (Table 4). The results for the BCLP yardstick showed that all centers except the centers in New Zealand (Fowler et al., 2019) had a mean score lower than 3, which indicates a good treatment result. In this systematic review the certainty of the evidence for outcomes of dental arch relationships, however, was very low and therefore the question remains whether any superiority of a treatment protocol exists for patients with complete BCLP, as is also the case for a treatment protocol for patients with complete UCLP (Nollet et al., 2005).

The evidence that the influence of the surgeon on the outcome is high was reported by Shaw et al. (Shaw et al., 1992) and reinforced by the results of the SCANDCLEFT trial (Rautio et al., 2017; Shaw and Semb, 2017). However, besides the influence of the surgeon, the influences of other surgical factors are still uncertain. The type and timing of lip and palatal closure are still a debate. Of the included 12 centers, except Center J with no surgical information, all the other 11 centers finished the lip repair before one year of age. Seven centers adopted the one-stage lip closure (Center A, B, E, F, G, I, K), and this surgery was performed as early as three months of age on average and the latest was performed at the mean age of 7.2 months. The other three centers (Center C, D, H) adopted a two-stage lip closure and the repairs were done before the age of 8 months. Center L undertook the primary lip repair at 3–6 months of age, but the surgical method was not indicated. Discussion on the timing of closure of the soft and hard palate is still continuing. Timing and technique of surgical closure of the palate may be related to midfacial growth (Guideline for cleft lip and palate. [richtlijn behandeling van patiënten met een schisis]). Of the 12 included centers, Center A, B, D (np-group), E, G, H and I adopted two-stage palatal surgery with early veloplasty and delayed hard palate repair. Center C also used a two-stage palatal surgery, but with an early hard palate repair and delayed veloplasty. The earliest palatal surgery was performed at 3 months of age, while the latest surgery was done at an average age of 9.9 years old. Center D (pp-group), F and K adopted the one-stage palatal repair at an age of 20–40 months, 6–8 months and 18 months respectively. Center L finished the palate repair at 9–12 months of age. The management of the alveolar cleft has lesser debate. The current used treatments for alveolar repair are secondary bone grafting and primary gingivoperiosteoplasty, while the former one is considered as the standard therapy in most centers (Wang et al., 2016). Except for the periosteoplasty group of Center D (periosteoplasty at 4 months and secondary bone graft at 11.5 years on average), the 5 centers (Center A, B, C, D, E) that provided information on surgical alveolar repair information, all performed secondary bone grafting around 8–12 years of age.

It takes a long time until growth has ceased to determine the final outcome of a treatment protocol for patients with a cleft (Berkowitz, 2013). The outcome assessed as the dental arch relationship is one outcome domain out of many such as speech, facial appearance, occlusion, upper airway function, cost effectiveness and burden of care and others. Many domains are related to the growth of which the final effect can only be evaluated after the growth is complete around the age of 18–19 years. The dental arch relationship reflects facial growth to some extent (Nollet et al., 2005). It is assessed in different stages of the patients’ growth and helps to judge the treatment effect of each stage. The judgement is based on dental casts, which are a standard record in orthodontic patients with a cleft and therefore often well accessible for outcome evaluation. Therefore evaluation of dental arch relationship should be a well-established way to judge the treatment outcome (Nollet et al., 2005).

The Huddart scoring system grades dental outcome by considering the bucco-palatal relationships in terms of frequency and severity of cross-bites in the anterior and buccal segments in order to evaluate maxillary constriction (Heidbuchel and Kuijpers-Jagtman, 1997). In patients with UCLP, it was found to be more reliable, objective and sensitive than the GOSLON and 5-year old Yardstick indices (Nollet et al., 2005). The advantages of the Huddart system are objectivity, relative simplicity, and no requirement for either anchor study models or a calibration course (Ma et al., 2017). A disadvantage of this scoring system is the fact that it does not score antero-posterior skeletal and vertical discrepancies and does not take into account incisor inclinations (Dobbyn et al., 2011). The Bauru-BCLP yardstick or BCLP yardstick as it was called later (Ozawa et al., 2011) is based on the GOSLON yardstick (Mars et al., 1987) and was developed to grade the dental arch relationship in BCLP. It assesses the dental arch relationship in terms of antero-posterior, transverse, and vertical discrepancies. The advantage of this scoring system over the Huddart system is that it takes greater account of the skeletal component. However, the use of the BCLP yardstick requires orthodontists who are experienced in treating patients with orofacial clefts (Bartzela et al., 2011). Though differences existed between the two grading systems, a high correlation was found in a study judging the two systems (Bartzela et al., 2011). To gain a better understanding of outcomes in patients with BCLP, it is important to combine the outcomes of both grading systems. Although most studies used the BCLP yardstick, the way data was reported was inconsistent amongst the included studies. Of the five studies using the BCLP Yardstick (Bartzela et al., 2010; Dissaux et al., 2016; Batra et al., 2018; Bittermann et al., 2018; Fowler et al., 2019), one presented the results as score categories 1 + 2, 3, 4 + 5 with the percentage of patients in each category (Dissaux et al., 2016). Another study used four categories with 1 + 2, 3, 4, 5 with the number and percentage of patients in each category (Batra et al., 2018). Two studies reported a detailed distribution of the scores with the number of patients (Bittermann et al., 2018; Fowler et al., 2019) and the remaining study only presented the mean BCLP scores (Bartzela et al., 2010). For a good comparison using the BCLP Yardstick as a categorization tool with five separate scores, the full distribution for all the categories should be reported in a consistent way. Meanwhile, the BCLP yardstick was developed for the primary and mixed dentition by Ozawa and Semb using a relatively small sample size. Afterwards, in 2011, Nijmegen, Gothenburg, Manchester, Bauru, and Oslo brought together 776 BCLP models to develop three yardsticks: 6-, 9-, 12-Year-Olds’ yardsticks, and they were named simply 6-, 9-, 12-Year-Olds’ BCLP yardsticks (Ozawa et al., 2011). So the Bauru-BCLP yardstick as originally reported, and the simply 6-, 9-, 12-Year-Olds’ BCLP yardsticks were not quite the same. We call for future studies to use the most recent version of the BCLP yardstick as described in Ozawa et al. (2011).

Of the three studies using the Huddart scoring system (Bartzela et al., 2011; Andlin Sobocki et al., 2012; Cassi et al., 2017), two articles presented the outcomes as the incisal segment, buccal segment and the total score (Bartzela et al., 2011; Andlin Sobocki et al., 2012), of which one separated the buccal segment scores into left and right segment (Andlin Sobocki et al., 2012) and the other presented the total buccal segment scores only (Bartzela et al., 2011). The remaining study presented the total Huddart score (Cassi et al., 2017). Similar to the use of the Bauru-BCLP Yardstick, when using the Huddart scoring system, the full set of scores of the incisal and buccal segment and the total scores should be reported. The comparison of treatment outcomes in patients with BCLP with the Huddart and/or Bauru-BCLP yardstick scores in this study is unfortunately not possible due to the above-mentioned heterogeneity and risk of bias.

Researchers in the field of cleft lip and palate are presented with a challenge. To gain scientific evidence, the sample size of clinical studies should increase. This means that multicenter studies are needed but this increases heterogeneity. A possible solution could be the development of clinical practice guidelines to diminish variability in treatment protocols between centers and hence enable future research with less heterogeneity between samples.

## Limitations

This systematic review has some limitations. First, the considered publications in this systematic review were all observational studies with a retrospective study design and therefore present a lower level of evidence. However, a well-designed observational study may provide some evidence when a randomized controlled clinical trial may not be possible to execute. Second, due to the heterogeneity and risk of bias of the included studies, no further synthesizing the results of the included studies into a meta-analysis was performed.

## Conclusions

Results for the dental arch relationship of studies in complete BCLP and possible determinants could not be synthesized due to high heterogeneity and risk of bias of the included studies. The quality of the evidence was very low. However, this study is meant to help and inspire clinicians and researchers in the field of clip and palate to start developing a general research framework to gain better data that in the end will improve patient care. Our recommendations for this framework are to agree upon a system of diagnostic (sub)phenotyping and a standard set of background data to be reported, and to develop a core outcome set using validated outcome tools. Clinical research for patients with BCLP should focus on a sound methodological design to enable evidence-based decision making to improve treatment for patients with BCLP and thereby hopefully their quality of life.
